# Pregnancy, Childbirth, Puerperium, Breastfeeding, and Sexuality in the World of Rare Diseases

**DOI:** 10.3390/healthcare11091351

**Published:** 2023-05-08

**Authors:** Raquel Rodríguez-Blanque, Juan Carlos Sánchez-García, Jonathan Cortés-Martín

**Affiliations:** 1 Research Group CTS1068, Andalusia Research Plan, Junta de Andalucía, 18016 Granada, Spain; rarobladoc@ugr.es (R.R.-B.); jcortesmartin@ugr.es (J.C.-M.); 2 School of Nursing, Faculty of Health Sciences, University of Granada, 18071 Granada, Spain; 3 Hospital Universitario Clínico San Cecilio, 18016 Granada, Spain

Rare diseases, also known as orphan diseases, are medical conditions that affect a small percentage of the population. In many cases, these diseases are chronic, debilitating, and often life-threatening. While each rare disease may affect only a small number of people, collectively, rare diseases are quite common, affecting an estimated 400 million people worldwide.

In recent years, there has been increasing attention focused upon rare diseases and their impact on individuals, families, and society. This increased awareness has led to improvements in diagnosis, treatment, and support for people with rare diseases, as well as increased research on these conditions.

Advances in genomic sequencing and other technologies have also helped identify the genetic basis of many rare diseases, opening up new possibilities for targeted therapies and personalized medicine. Additionally, patient advocacy groups and organizations have been instrumental in raising awareness, promoting research, and supporting individuals and families affected by rare diseases.

A rare disease is a group of pathologies, and their main feature is their low prevalence in the population [1]. In order to be classified as rare, the disease must affect less than 1 in 2000 live births [2]. There are approximately 8000 rare diseases today. Eighty percent of these pathologies have a genetic origin, and the vast majority of the remaining 20% have a metabolic origin [3].

The main problems faced by patients diagnosed with one of these diseases are chronicity, degenerative nature, high disabling potential, and high mortality rates [1].

In addition, the delay in diagnosis poses a major problem for this field, a patient may take on an average of 4–5 years to obtain a diagnosis. Approximately 20% of patients take approximately 10 years to obtain a diagnosis [3]. In most cases, there is a delay in diagnosis due to widespread ignorance in the field of rare diseases.

Although the advances of the scientific community on these diseases are undoubted, to date, 42.68% of people with these pathologies do not have treatment, or if they do, it is inadequate [3].

Rare diseases can affect various aspects of human health, including sexual development and function. However, the specific impact of rare diseases on sexuality can vary greatly depending on the disease and the patient [4].

Some rare genetic disorders, such as Klinefelter syndrome, Turner syndrome, and XYY syndrome, can affect sexual development and result in differences in sexual characteristics and/or function. For example, individuals with Klinefelter syndrome may have reduced testosterone levels, which can lead to decreased libido and erectile dysfunction [5].

Other rare diseases may not directly affect sexual development or function, but they may impact sexual health in other ways. For example, people with spinal muscular atrophy (SMA) may have physical limitations that affect sexual activity, such as difficulties with positioning or movement. Similarly, people with cystic fibrosis may experience infertility due to the abnormal development of the reproductive tract [6].

Due to the particularity of these conditions, information on their impact on sexuality may be limited. 

Considering the impact of this type of pathology on pregnancy, childbirth, puerperium, and the implantation and maintenance of breastfeeding, there are great differences depending on the specific disease.

Preconception counseling is essential in women affected by this type of pathology in order to assess the potential risks and optimize their health before conception [7].

Depending on the specific rare disease, pregnancy may carry an increased risk of complications, such as preterm delivery, growth retardation, preeclampsia, and fetal anomalies. Close follow-up by a team of healthcare professionals, including a specialist in the rare disease, is recommended [8].

Delivery planning should take into account any potential complications related to the rare disease, such as the need for cesarean delivery or specialized neonatal care. The puerperium may also be complicated by the rare disease, such as the risk of the exacerbation of symptoms or medication management considerations [9].

Ehlers–Danlos syndrome manifests a connective tissue disorder that may increase the risk of the premature rupture of membranes, cervical incompetence, and uterine prolapse during pregnancy [10].

In Marfan syndrome, the risk of aortic dissection and rupture during pregnancy may be increased due to connective tissue disorders [11].

Mitochondrial disorders can affect several organs, including the heart and muscles, leading to an increased risk of fetal growth restriction, preterm delivery, and stillbirth [12].

Systemic lupus erythematosus can increase the risk of preeclampsia, preterm delivery, and fetal loss [13].

Overall, it is important for women with rare diseases to work closely with their healthcare professionals to optimize their health and manage any potential complications during pregnancy, delivery, and the postpartum period.
